# Comparison of accuracy between FSL’s FIRST and Freesurfer for caudate nucleus and putamen segmentation

**DOI:** 10.1038/s41598-017-02584-5

**Published:** 2017-05-25

**Authors:** Gabor Perlaki, Reka Horvath, Szilvia Anett Nagy, Peter Bogner, Tamas Doczi, Jozsef Janszky, Gergely Orsi

**Affiliations:** 1 MTA-PTE Clinical Neuroscience MR Research Group, Pecs, 7623 Hungary; 2 Pecs Diagnostic Centre, Pecs, 7623 Hungary; 3 Department of Neurology, University of Pecs, Medical School, Pecs, 7623 Hungary; 4 MTA-PTE Neurobiology of Stress Research Group, Pecs, 7624 Hungary; 5 Department of Radiology, University of Pecs, Medical School, Pecs, 7624 Hungary; 6 Department of Neurosurgery, University of Pecs, Medical School, Pecs, 7623 Hungary; 7 Centre for Neuroscience, University of Pécs, Pécs, 7623 Hungary

## Abstract

Although several methods have been developed to automatically delineate subcortical gray matter structures from MR images, the accuracy of these algorithms has not been comprehensively examined. Most of earlier studies focused primarily on the hippocampus. Here, we assessed the accuracy of two widely used non-commercial programs (FSL-FIRST and Freesurfer) for segmenting the caudate and putamen. T1-weighted 1 mm^3^ isotropic resolution MR images were acquired for thirty healthy subjects (15 females). Caudate nucleus and putamen were segmented manually by two independent observers and automatically by FIRST and Freesurfer (v4.5 and v5.3). Utilizing manual labels as reference standard the following measures were studied: Dice coefficient (*D*), percentage volume difference (*PVD*), absolute volume difference as well as intraclass correlation coefficient (ICC) for consistency and absolute agreement. For putamen segmentation, FIRST achieved higher *D*, lower *PVD* and higher ICC for absolute agreement with manual tracing than either version of Freesurfer. Freesurfer overestimated the putamen, while FIRST was not statistically different from manual tracing. The ICC for consistency with manual tracing was similar between the two methods. For caudate segmentation, FIRST and Freesurfer performed more similarly. In conclusion, Freesurfer and FIRST are not equivalent when comparing to manual tracing. FIRST was superior for putaminal segmentation.

## Introduction

Magnetic resonance imaging (MRI)-based delineation of brain regions is an important technique that plays an expanding role in neuroscience research. MRI-based segmentation studies have revealed volumetric changes related to a wide range of factors including various functional, behavioral, demographic, nutritional, environmental and biological attributes^1–9^ as well as the presence of several neurological and psychiatric conditions^10–14^. Besides volumetric investigations, structural MRI-based region delineation can be used to extract regional MR or nuclear medicine imaging parameters (e.g. diffusion, relaxation parameters, tracer uptake)^15–17^.

Traditionally, brain regions are segmented manually and manual segmentation is considered the gold standard approach even today. However, this simple method is subjective, extremely time-consuming, laborious and human resource intensive and thus unfeasible for large MRI data sets^18, 19^. To overcome these limitations, several automated segmentation tools were proposed, including the widely used non-commercial FSL-FIRST and Freesurfer methods. Despite both of these methods are well-published and validated by their developers^20, 21^, relatively few studies discussed the comparison of their accuracy and most of these studies focused primarily on the segmentation of hippocampus^22–27^. These results cannot be generalized to other brain regions. Moreover, both FSL and Freesurfer are actively developed, which highlights the importance of revalidating the usefulness of these packages from time to time.

Our aim was to compare the accuracy of automated segmentation of the caudate and putamen by FIRST and Freesurfer, using manual tracing as reference standard. These striatal structures were chosen because of their importance in a variety of diseases including Parkinson’s disease^28^, Huntington’s disease^29^, obsessive-compulsive disorder^30^, Alzheimer’s disease^31, 32^, primary focal dystonia^33^, attention-deficit hyperactivity disorder^34^, depression^35^ and schizophrenia^36^ and because their segmentation is challenging due to the fact that MR image intensities alone cannot be used to successfully distinguish them from adjacent brain structures^21^.

## Results

### Dice coefficients

**Figure 1:**
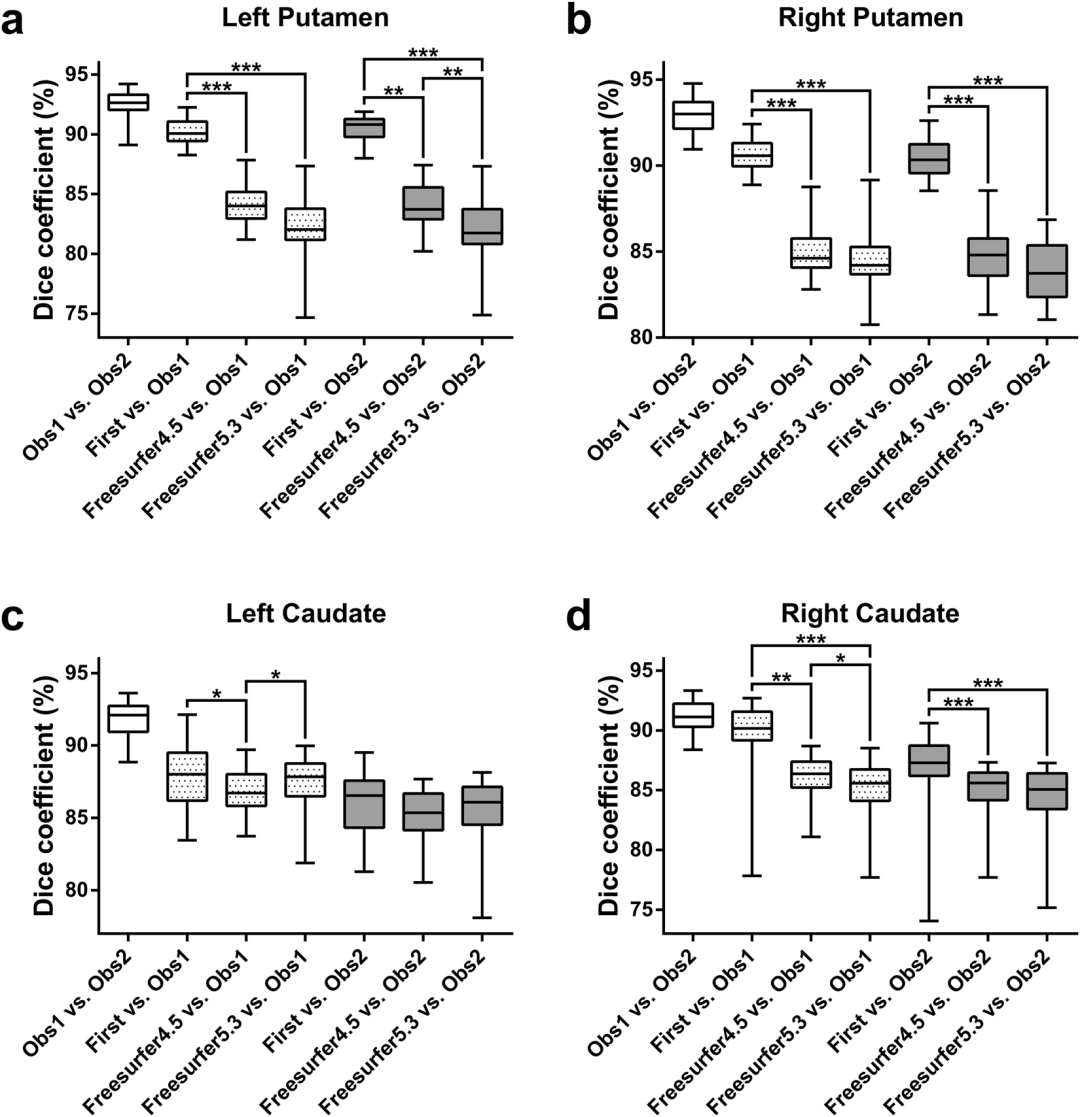
Spatial overlap (*D*) between the segmentation methods. The dotted and the gray boxes show the spatial overlap relative to the manual tracing by Observer 1 and Observer 2, respectively. Significant differences among the three automated methods when compared to manual tracing are marked by asterisks (*P < 0.01, **P < 0.001, ***P < 0.0001; post-hoc Dunn’s multiple comparisons test). Whiskers are set at minimum and maximum, the horizontal line marks the median, whereas box indicates the interquartile range (25–75%).

The Friedman test revealed significant differences in Dice coefficient (*D*) among the automated segmentation methods in all four brain structures (P < 0.01). Irrespective of using Observer 1 or Observer 2 as reference, post-hoc Dunn’s tests indicated significantly higher *D* for FIRST than both versions of Freesurfer, bilaterally in the putamen and in the right caudate (Fig. 1). Other significant differences in *D* were not consistent (depended on which observer was used as reference). If FIRST was run in Freesurfer space (on orig.mgz) and the segmentation was mapped back into native space, the results for Putamen were unchanged, but the consistency of higher *D* for FIRST in the right caudate was diminished and FIRST even performed consistently worse in the left caudate than Freesurfer 5.3 (Supplementary Fig. S1).

### Percentage volume differences

**Figure 2:**
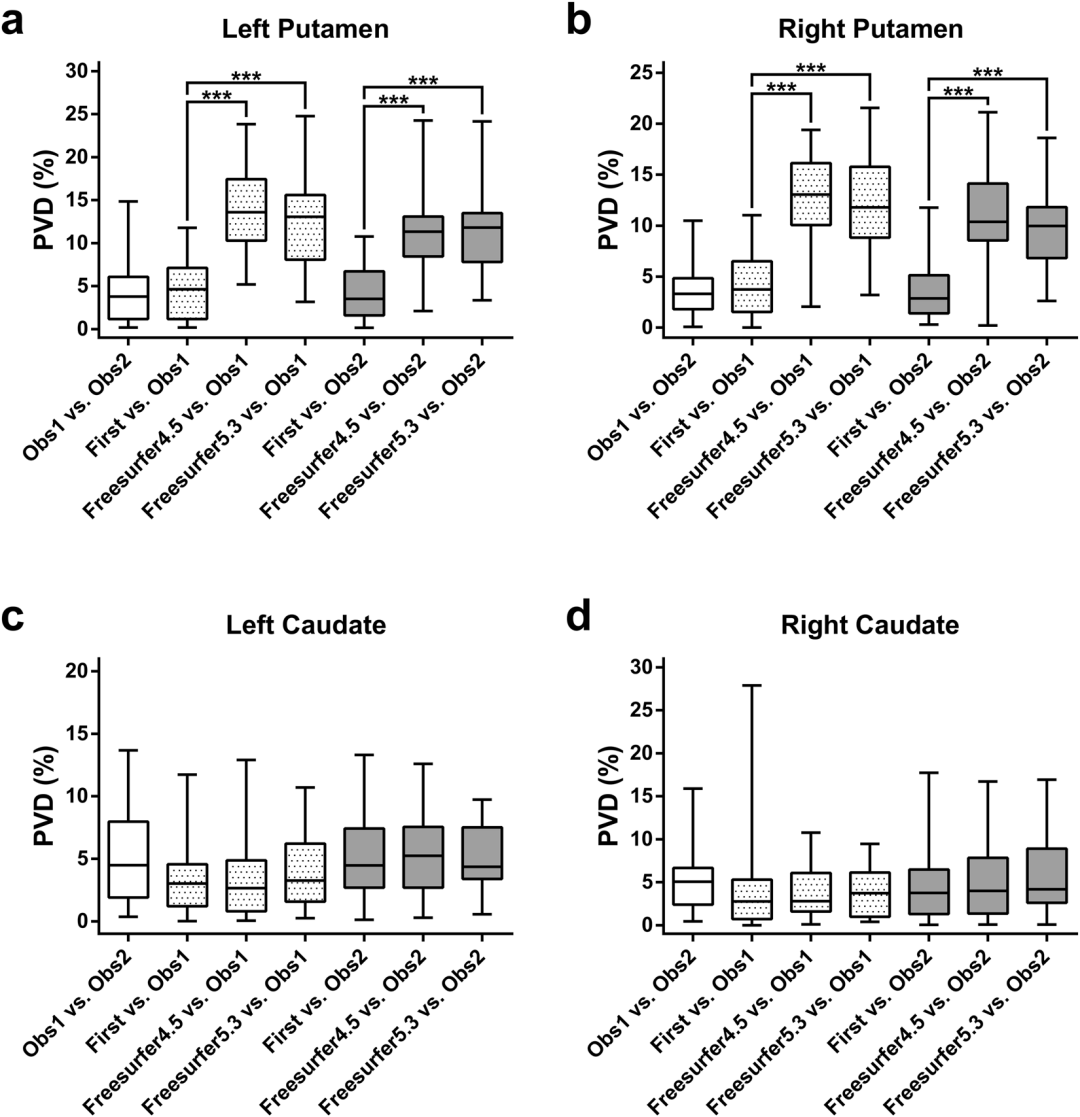
Percentage volume difference (*PVD*) between the segmentation methods. The dotted and the gray boxes show the percentage volume difference relative to the manual tracing by Observer 1 and Observer 2, respectively. Significant differences among the three automated methods when compared to manual tracing are marked by asterisks (*P < 0.01, **P < 0.001, ***P < 0.0001; post-hoc Dunn’s multiple comparisons test). Whiskers are set at minimum and maximum, the horizontal line marks the median, whereas box indicates the interquartile range (25–75%).

The Friedman test revealed significant differences in percentage volume difference (*PVD*) in the putamen bilaterally (P < 0.0001). Post-hoc Dunn’s tests indicated that *PVD* values were significantly lower for FIRST than both versions of Freesurfer, bilaterally in the putamen (Fig. 2).

### Volumetric differences

The Friedman test revealed significant volumetric differences among the segmentation methods in all four brain structures (P < 0.0001). Post-hoc Dunn’s test indicated no significant differences between FIRST and manual tracing in any of the four brain structures. Both versions of Freesurfer resulted in significantly greater putaminal volumes than the other methods. The significantly higher caudal volumes by Freesurfer were not consistent, depending on which version of Freesurfer was compared to which particular method. Observer 2 delineated significantly smaller caudates than Observer 1. No significant volumetric differences were detected between Freesurfer 4.5 and Freesurfer 5.3 (Table 1).

**Table 1:** Statistical comparison of the volumes resulting from different segmentation approaches.

Segmentations	Left Putamen		Right Putamen		Left Caudate		Right Caudate	
	Median [range]	Dunn’s test	Median [range]	Dunn’s test	Median [range]	Dunn’s test	Median [range]	Dunn’s test
Observer 1	5278 [4191–6264]	ns	5226 [4233–6486]	ns	3779 [3082–5009]	P = 0.0045	3958 [3135–5269]	P = 0.0095
Observer 2	5544 [4294–6395]		5371 [4146–6453]		3648 [3062–4828]		3805 [2992–5293]	
First	5328 [4196–6587]	ns	5386 [4282–6943]	ns	3739 [3208–5069]	ns	3821 [2791–5122]	ns
Observer 1	5278 [4191–6264]		5226 [4233–6486]		3779 [3082–5009]		3958 [3135–5269]	
First	5328 [4196–6587]	ns	5386 [4282–6943]	ns	3739 [3208–5069]	ns	3821 [2791–5122]	ns
Observer 2	5544 [4294–6395]		5371 [4146–6453]		3648 [3062–4828]		3805 [2992–5293]	
Freesurfer 4.5	5983 [4960–7826]	P < 0.0001	5848 [4938–7059]	P < 0.0001	3812 [3212–5032]	ns	3970 [3064–5076]	ns
Observer 1	5278 [4191–6264]		5226 [4233–6486]		3779 [3082–5009]		3958 [3135–5269]	
Freesurfer 4.5	5983 [4960–7826]	P < 0.0001	5848 [4938–7059]	P < 0.0001	3812 [3212–5032]	P < 0.0001	3970 [3064–5076]	ns
Observer 2	5544 [4294–6395]		5371 [4146–6453]		3648 [3062–4828]		3805 [2992–5293]	
Freesurfer 5.3	5732 [5005–7690]	P < 0.0001	5602 [4644–7304]	P < 0.0001	3800 [3245–4983]	ns	4020 [3323–5452]	ns
Observer 1	5278 [4191–6264]		5226 [4233–6486]		3779 [3082–5009]		3958 [3135–5269]	
Freesurfer 5.3	5732 [5005–7690]	P < 0.0001	5602 [4644–7304]	P < 0.0001	3800 [3245–4983]	ns	4020 [3323–5452]	P < 0.0001
Observer 2	5544 [4294–6395]		5371 [4146–6453]		3648 [3062–4828]		3805 [2992–5293]	
First	5328 [4196–6587]	P < 0.0001	5386 [4282–6943]	P < 0.0001	3739 [3208–5069]	ns	3821 [2791–5122]	ns
Freesurfer 4.5	5983 [4960–7826]		5848 [4938–7059]		3812 [3212–5032]		3970 [3064–5076]	
First	5328 [4196–6587]	P < 0.0001	5386 [4282–6943]	P = 0.0002	3739 [3208–5069]	ns	3821 [2791–5122]	P = 0.0015
Freesurfer 5.3	5732 [5005–7690]		5602 [4644–7304]		3800 [3245–4983]		4020 [3323–5452]	
Freesurfer 4.5	5983 [4960–7826]	ns	5848 [4938–7059]	ns	3812 [3212–5032]	ns	3970 [3064–5076]	ns
Freesurfer 5.3	5732 [5005–7690]		5602 [4644–7304]		3800 [3245–4983]		4020 [3323–5452]	

Median [range] of volumes are reported in mm^3^; ns = statistically not significant (P > 0.01).

### Intraclass correlation coefficients

Intraclass correlation coefficients (ICCs) between the volumes from different segmentation approaches are shown in Table 2. The consistency and the absolute agreement among the two observers and FIRST were good to excellent for all four brain structures (0.823–0.946). Consistency of the observers with both versions of Freesurfer was also good to excellent (0.864–0.932) for all four structures, except for the left putamen segmented by Freesurfer 5.3 (ICC = 0.766 and ICC = 0.706 for the consistency with Observer 1 and Observer 2, respectively). Absolute agreement of the observers with both versions of Freesurfer was good to excellent for the caudate (0.857–0.925), while mainly in the non-acceptable range for the putamen (0.473–0.670). The consistency and absolute agreement between Freesurfer versions were good to excellent for all four structures (0.867–0.989).

**Table 2:** Intraclass correlation coefficients between the volumes calculated from different segmentation approaches.

Segmentations	Left Putamen		Right Putamen		Left Caudate		Right Caudate	
	Absolute agreement	Consistency	Absolute agreement	Consistency	Absolute agreement	Consistency	Absolute agreement	Consistency
Observer 1	0.823	0.843	0.903	0.921	0.860	0.895	0.875	0.911
Observer 2								
First	0.861	0.867	0.886	0.946	0.921	0.920	0.887	0.893
Observer 1								
First	0.883	0.882	0.910	0.918	0.873	0.90	0.906	0.914
Observer 2								
Freesurfer 4.5	0.473	0.864	0.478	0.904	0.911	0.913	0.917	0.914
Observer 1								
Freesurfer 4.5	0.573	0.879	0.592	0.910	0.862	0.93	0.865	0.897
Observer 2								
Freesurfer 5.3	0.475	0.766	0.538	0.889	0.916	0.913	0.925	0.930
Observer 1								
Freesurfer 5.3	0.516	0.706	0.670	0.923	0.889	0.925	0.857	0.932
Observer 2								
First	0.533	0.853	0.638	0.889	0.941	0.950	0.879	0.883
Freesurfer 4.5								
First	0.497	0.710	0.705	0.890	0.942	0.940	0.883	0.914
Freesurfer 5.3								
Freesurfer 4.5	0.867	0.874	0.943	0.950	0.983	0.989	0.953	0.964
Freesurfer 5.3								

## Discussion

This study examined the reliability of two popular non-commercial automatic programs (FSL-FIRST and Freesurfer) for segmenting the caudate and putamen in a group of normal subjects, using manual segmentations by two independent observers as reference. Previous studies comparing Freesurfer with FIRST have focused primarily on the hippocampus in healthy and various patient groups (see Introduction). Only one study, to our knowledge, attempted to compare the reliability of Freesurfer and FIRST relative to manual tracing in the caudate and putamen^37^, but it did not report spatial overlap of the automated segmentations with manual tracing and their results may be suboptimal due to including both healthy subjects and psychiatric patients in the same statistics and using MRI measurements which were not standardized across the small number of subjects (N = 20).

**Figure 3:**
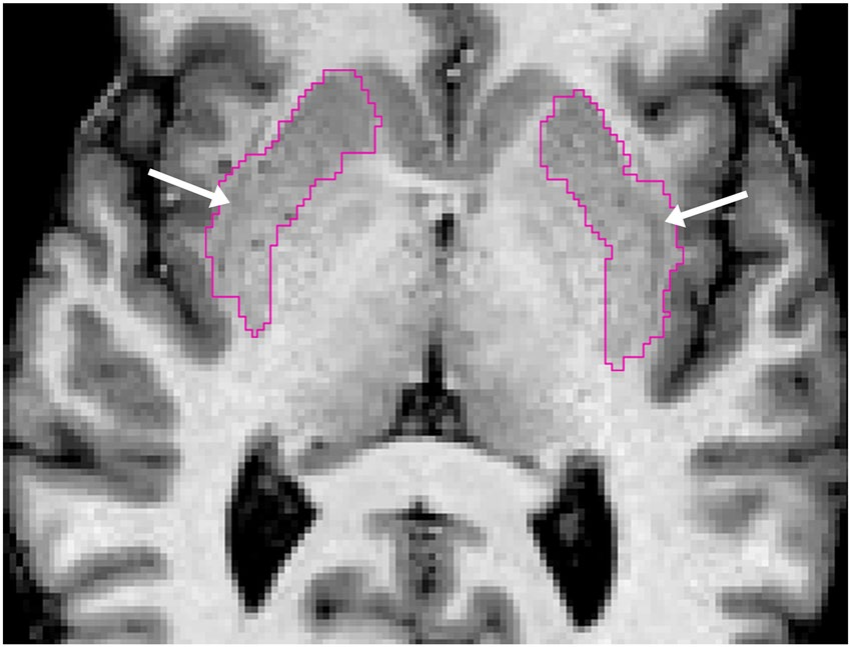
Figure illustrating the putamen segmentation by Freesurfer 5.3 in one of our healthy subjects. Pink indicates the outline of left and right putamen segmentations and white arrows point to the mis-segmented area intruding into the claustrum.

We found that FIRST was superior to both versions of Freesurfer in segmenting the putamen as demonstrated by higher *D*, lower *PVD* and higher ICC for absolute agreement with manual tracing as well as non-significant volumetric differences compared with manual tracing. The higher absolute agreement (ICC) for FIRST is consistent with the results of Nugent *et al*., but our ICC values were generally higher^37^. Both versions of Freesurfer overestimated the putamen bilaterally, which seems to be sufficiently systematic as demonstrated by acceptable to excellent ICC for consistency with manual tracing. In contrast to Nugent *et al*., we found putaminal volume overestimation only for the Freesurfer, but not for FIRST. Another study comparing Freesurfer with IBASPM in HIV-infected patients also found that FreeSurfer systematically overestimated the putamen^38^. Slice by slice visual inspection of the automatic putamen segmentations – by both observers together – exposed that putaminal overestimation by Freesurfer is probably due to the partial inclusion of the claustrum, especially around the posterior-inferior quadrant of the putamen (Fig. 3). This segmentation error could be clearly identified in all of our subjects and even in Freesurfer’s sample subject “Bert” using both versions of Freesurfer, while it was visually absent for all of the putamen segmentations by FIRST. Therefore, one may speculate that this issue is probably related to the default segmentation model/method underlying Freesurfer rather than our sample. This suspicion is further strengthened by the results of Dewey *et al*., who found that the vast majority of Freesurfer putaminal overestimation was attributable to the inclusion of external capsule and claustrum in HIV-infected patients as well. In addition, to characterize the bias behind putaminal overestimation by Freesurfer more objectively, 3D shape analyses were performed between Freesurfer and manual tracing (see Supplementary Methods). The results of shape analyses also confirmed that Freesurfer tended to inflate putamen, intruding into the claustrum. A representative image of the putaminal surface overestimation by Freesurfer 5.3 as compared to Observer 2 is shown in Supplementary Fig. S2.

Although the segmentation performance of FIRST was found to be better for the putamen according to most accuracy measures, the ICC for consistency with manual tracing was similar between Freesurfer and FIRST, suggesting that these automated methods give similar results when correlating the volumetric data with other variables (e.g. age) or comparing the volumes between two groups of subjects^39^. Only the ICC for consistency with manual tracing in the left putamen segmented by Freesurfer 5.3 was markedly lower, but still in an acceptable range (>0.7). However, when absolute putaminal volume data are of interest or the automatic segmentation is used to delineate putamen for quantitative data extraction (e.g. diffusion, relaxation parameters, PET/SPECT tracer uptake), where decreased spatial accuracy (i.e. due to size or shape differences or spatial location shifts) may have significant effects on the extracted measures, then FIRST seems to be a better choice.

For caudate segmentation, the automated methods performed more similarly. Freesurfer 4.5, Freesurfer 5.3 and FIRST had comparable ICC both for consistency and absolute agreement with manual tracing as well as similar *PVD* values. Although, it initially seemed that *D* was lower for both versions of Freesurfer in the right caudate, we showed that these small differences between Freesurfer and FIRST may be a consequence of methodological issues (i.e. Freesurfer does not segment in the native space and Freesurfer segmentation must be mapped back into native space for the computation of *D*). Future studies should be aware that small, but significant differences in *D* may be driven by these methodological differences. Results of some earlier studies may also be affected. In certain comparisons (i.e. Freesurfer 4.5 vs. Observer 2 for the left caudate; First vs. Freesurfer 5.3 and Freesurfer 5.3 vs. Observer 2 for the right caudate) Freesurfer resulted in significantly greater caudal volumes, but not consequently in all comparisons as in case of the putamen. Nevertheless these overestimations were quite small (3.4–5.4% overestimation in the mean volumes), which were not manifested in decreased absolute agreement ICC values for the Freesurfer. One of our observers was more liberal as demonstrated by the very small (<4% in the mean volumes), but significant volume differences bilaterally in the caudate. However, absolute ICC values between the two observers were in a good range, suggesting that this consequent difference is relatively small compared to the inter-subject volume differences. Our results also suggest that even experienced observers may produce highly consistent, but systematically different segmentations, which emphasize the importance of using more than one human rater as reference for validation purposes.

In conclusion, Freesurfer and FIRST are not equivalent when compared to manual tracing, especially for the segmentation of putamen, where FIRST exhibits higher performance. However, since consistency was similar, putamen segmentation by Freesurfer can also be recommended depending on the scope of the study.

## Methods

### Subjects

Thirty healthy, young, Caucasian subjects (15 females; mean age: 23.0 ± 2.7, range: 19–29 years) without any known disease were included. All subjects got detailed information about the investigation and gave written informed consent prior to the examination. The study was approved by the local ethical committee of the University of Pécs (4326.316-2899/KK14/2011.-2011.12.27.) and was performed in accordance with the ethical standards laid down in the 1964 Declaration of Helsinki and its later amendments.

### Magnetic resonance imaging

All subjects were scanned on the same 3 T MRI scanner (MAGNETOM Trio a Tim System, Siemens AG, Erlangen, Germany) with a 12-channel head coil. A high-resolution T1-weighted image was acquired for each subject using a strict standardized protocol (3D MPRAGE sequence; TR/TI/TE = 2530/1100/3.37 ms; Flip Angle = 7°; 176 sagittal slices; slice thickness = 1 mm; FOV = 256 × 256 mm^2^; matrix size = 256 × 256; receiver bandwidth = 200 Hz/pixel). Images were visually inspected in order to confirm appropriate image quality and to exclude subjects with visible brain abnormalities.

### Manual segmentation

Manual segmentation was done by two observers (G.O. and G.P., each with 8 years of experience in human brain MRI data processing and segmentation).

The segmentation of the dorsal striatum was carried out on two identical workstations using 24″ monitors with 5^th^ generation AMVA panels calibrated to 120 cd/m^2^, 6500 K and gamma of 2.2 (resulting in contrast ratio over 3000:1).

3DSlicer 4.3.1 (r22599) was used for the manual tracing on both workstations in 64 bit Linux environment.

Putamen segmentation was performed on successive axial slices starting from the uppermost slice, where the putamen is seen (superior border). Coronal images were used to verify and correct the borders of the structure, especially the inferior margin. Inferior border was formed by the anterior commissure, the anterior perforated substance and lateral lenticulostriatal artery. Medial border was defined by the internal capsule (anterior limb), nucleus accumbens, globus pallidus (lateral medullary lamina) and internal capsule (posterior limb), while lateral border was formed by the external capsule. Anterior and posterior borders were defined by the internal capsule (anterior and posterior limbs, respectively).

Caudate segmentation was also performed on axial slices starting on the uppermost slice on which caudate is visible (at the lateral border of the lateral ventricle). Inferior border (anterior to posterior): nucleus accumbens, anterior commissure, internal capsule (anterior limb), stria terminalis. Medial border was defined by the lateral ventricle and nucleus accumbens (anteroventral part of the caudate), while lateral border was defined by the internal capsule (anterior and posterior limbs) and the centrum semiovale (at the most dorsal part of the caudate nucleus). Anterior border was also defined by the lateral ventricle. The caudate was traced along the edge of the posterior horns of the lateral ventricle and was terminated at the most posterior part of the caudate (vertical portion), excluding the tail. Coronal images were also used to correct the boundaries of delineation and were of utmost importance to distinguish the structure from nucleus accumbens.

### Automated segmentation by FIRST

After reorienting the sagittal MR images to match the orientation of the MNI152 standard template image, the segmentation of caudate and putamen was performed with FSL-FIRST (FSL’s build: 507) initiated by the “run_first_all” script using default settings. The technical details of the FIRST algorithm were described previously^20^. Briefly, the images were initially registered to the MNI152 standard space template in a two-stage process, where the first stage was a whole-brain and the second stage was a subcortical-weighted 12 degrees of freedom linear fit. The registration was visually checked for each subject. The inverse of this transformation was applied to the segmentation model in order to bring it into the native space of the original (non-interpolated) T1-weighted image, where the segmentation was performed. FIRST uses Bayesian shape and appearance models constructed from a training set of manually segmented T1-weighted images provided by the Center for Morphometric Analysis (CMA), MGH, Boston. Based on the learned models, FIRST searched for the most probable shape instance of each subcortical structure being segmented, given the observed intensities from the input image. Surface meshes of the subcortical structures were converted to boundary corrected volumetric representations. For the boundary correction, the “auto” option was chosen, which is the default behavior of the “run_first_all” script. Finally, the successful segmentations of left and right caudate and putamen were visually verified and their masks were extracted into separate files from the single image containing the labels of all the 15 segmented subcortical structures (*output_name*_all_fast_firstseg.nii.gz).

### Automated segmentation by Freesurfer

Automated segmentations of the caudate and putamen were performed by two different versions of the Freesurfer (v4.5 and v5.3). Each image was processed on exactly the same computer (Intel Core i5-3570 based workstation) and operating system (Linux Mint 17. 64-bit), which is important because Freesurfer used on different workstations and operating systems may lead to different results^40^. The images were processed by running the “recon-all” script using the default analysis settings. Technical details of the automated subcortical segmentation stream are described in prior methodological publications^21, 41^ and on FreeSurferWiki page (https://surfer.nmr.mgh.harvard.edu/fswiki). Talairach transformation and the removal of non-brain tissues were visually verified and error correction was performed when necessary, based on the recommended workflow available at the FreeSurferWiki website. However, it should also be noted that no manual editing was performed on the final segmentation labels to ensure that no bias towards manual delineation is introduced. In order to make spatial comparisons with the manually segmented labels, the subcortical segmentation output of Freesurfer (aseg.mgz) was transformed back to the native space of the original MPRAGE image by using Freesurfer library function “*mri_label2vol”*. After that, the native space segmentations were reoriented to match the orientation of the MNI152 standard template images. Finally, the masks of left and right caudate and putamen were extracted into separate files. It should be noted that mapping the segmented labels back into native space was only necessary for the calculation of spatial overlap (i.e. Dice coefficient). For other accuracy measures, the partial volume corrected volume estimates in the aseg.stats Freesurfer file were used to get more precise results.

### Statistical measures

Statistical analyses were performed using SPSS 20.0 (IBM Corp., Armonk, NY) and GraphPad Prism 6.01 software.

Spatial overlap (Dice coefficient, *D*) and percentage volume difference (*PVD*) were calculated among the different segmentations according to the following equations:

1 $$D=100\times \frac{{\rm{V}}({\rm{A}}\cap {\rm{B}})}{\frac{{\rm{V}}({\rm{A}})+{\rm{V}}({\rm{B}})}{2}}$$

2 $$PVD=100\times \frac{|{\rm{V}}({\rm{A}})-{\rm{V}}({\rm{B}})|}{\frac{{\rm{V}}({\rm{A}})+{\rm{V}}({\rm{B}})}{2}}$$

where V is the volume function, ∩ is the intersection operation, A and B are the segmentations by two different methods.

The maximal value of *D* is 100, indicating perfect overlap between the two segmentations. Decreasing *D* indicates less overlap. The minimal value of *PVD* is zero, indicating equal volume of the two segmentations. Increasing *PVD* indicates greater volume difference between the two segmentations.

The segmentation accuracy of the automated methods was assessed by comparing with manual tracing as reference standard. Significant differences in *D* and *PVD* among the three automated methods were assessed using Friedman test followed by two-tailed Dunn’s test corrected for multiple comparisons. Without assuming that either observer is more accurate, *D* and *PVD* values were calculated twice, considering Observer 1 or Observer 2 as the reference, and statistical tests were also run twice accordingly.

In addition to the accuracy measures, significant volume differences were assessed using Friedman test followed by two-tailed Dunn’s test corrected for multiple comparisons. Intraclass correlation coefficients (ICCs) were also calculated to assess the consistency (i.e. systematic differences are irrelevant) and absolute agreement (i.e. systematic differences are relevant) between the volumes resulting from different segmentation approaches. Two-way mixed model was selected and ICCs were obtained for single measures. As a rule of thumb, ICC ≥ 0.9 was considered to be excellent, 0.9 > ICC ≥ 0.8 was considered to be good and 0.8 > ICC ≥ 0.7 was considered to be acceptable.

Each statistical analysis was based on all subjects included in the study (n = 30). Results were considered significant at P ≤ 0.01 for all statistical tests.

## Methodological Considerations

We used manual segmentation as our reference standard and although manual segmentation is commonly used as the reference technique for assessing the performance of automatic segmentation techniques, it is subjective and it is unknown how well manual tracing represents the true boundaries of the segmented structures. However, the strength of our article in contrast to some earlier papers is that manual tracing was performed by two independent observers to obtain those robust results which are relatively independent of the observer itself.

To calculate Dice coefficients, Freesurfer segmentations had to be transformed from Freesurfer space back to the native space of the original MPRAGE image, which may result in slight alterations due to resampling. Although, this step was performed consistently with earlier studies^25, 38^ as recommended by Freesurfer developers (https://surfer.nmr.mgh.harvard.edu/fswiki/FsAnat-to-NativeAnat), our results show that mapping the raw T1-image into a non-native space, segmenting on that interpolated image and mapping back into native space may have significant impact on calculated Dice values (i.e. caudate).

The results could be specific to the particular MRI acquisition parameters used. However, since our MPRAGE protocol was set up based on the recommended morphometry protocols for optimal FreeSurfer reconstruction (available at: https://surfer.nmr.mgh.harvard.edu/fswiki/), we suggest that the lower performance of FreeSurfer is not attributable to the current acquisition protocol.

We studied healthy subjects in a narrow age range, which limits the generalizability of our results. However, using this healthy young sample, it is unlikely that our reliability measurements are limited by the atlases underlying Freesurfer and FIRST consisting of a wide range of demographics^42^. Even so, certain reliability measures were rather low in some cases, which may be even worse in a sample less representative of the general population.

It should be noted, that our results may only apply to the versions of Freesurfer and FIRST tested.

### Disclosure on restrictions of data availability

The transfer/disclosure of raw MRI images to 3rd party had not been approved in the ethical approval obtained for this study (issued by the local ethical committee of the University of Pécs 4326.316-2899/KK14/2011.-2011.12.27.).

## Electronic supplementary material

Supplementary Information

Dataset 1
