# Supplementary material for: Comparison of accuracy between FSL’s FIRST and Freesurfer for caudate nucleus and putamen segmentation
Source: Sci Rep. 2017 May 25;7:2418. doi: 10.1038/s41598-017-02584-5 (PMC5445091; doi:10.1038/s41598-017-02584-5)
Supplement: Supplementary file 1 — Supplementary Information [file 41598_2017_2584_MOESM1_ESM.pdf]

Supplementary Information

Comparison of accuracy between FSL's FIRST and Freesurfer for caudate nucleus and putamen segmentation

Gabor PERLAKI PhD[a,b,f], Reka HORVATH MD PhD[c], Szilvia Anett NAGY MS[b,d], Peter BOGNER MD PhD[e], Tamas DOCZI MD PhD[a,b,f], Jozsef JANSZKY MD PhD[a,c], Gergely ORSI PhD[a,b,f,*]

[a]MTA-PTE Clinical Neuroscience MR Research Group, H-7623 Pecs, Hungary
[b]Pecs Diagnostic Centre, H-7623 Pecs, Hungary
[c]Department of Neurology, University of Pecs, Medical School, H-7623 Pecs, Hungary
[d]MTA-PTE Neurobiology of Stress Research Group, H-7624 Pecs, Hungary
[e]Department of Radiology, University of Pecs, Medical School, H-7623 Pecs, Hungary
[f]Department of Neurosurgery, University of Pecs, Medical School, H-7623 Pecs, Hungary

[*]Corresponding author:
Gergely ORSI, PhD
Ret str. 2.
MTA-PTE Clinical Neuroscience MR Research Group
H-7623
Pecs
Hungary
phone/fax: +36/72/242312
email: gergo.orsi@gmail.com

Supplementary Methods

Systematic shape differences between Freesurfer and manual tracing were calculated for the putamen by using FSL tools [1]. The binary putamen segmentations by Freesurfer and manual tracing were transformed to the 1mm MNI152 standard space for each subject by using the subcortical-weighted 12 degrees of freedom registration matrix calculated during FIRST segmentation. The output datatype was chosen as float for the transformed labels. All transformed label images were then averaged and the average image was thresholded at 0.5 to get a binarized average shape image. A boundary image was formed by taking the average shape image, eroding it by using a 3D kernel of 3x3x3 voxels and subtracting the eroded version from the original. The signed distance of the average boundary points to MNI space putamen labels (binarized by thresholding at 0.5) were calculated for each subject and for each segmentation method. To assess shape differences causing systematic putamen overestimation by Freesurfer relative to manual tracing, permutation-based nonparametric paired t-test was run (5000 permutations) on the calculated distance maps [2]. Results were considered significant for $P < 0.05$, corrected for multiple comparisons at voxel-level.

Supplementary Figures

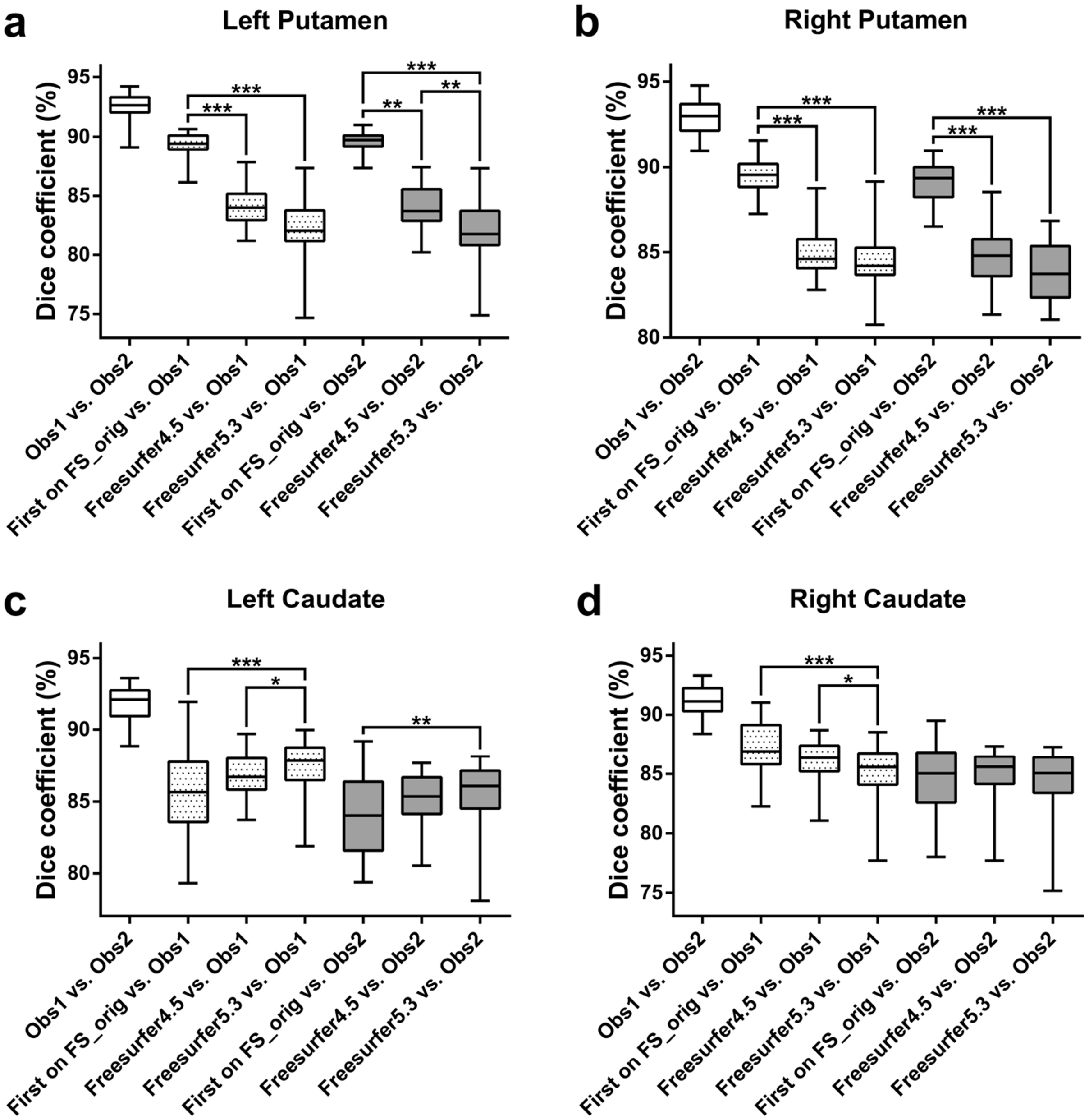

Supplementary Figure S1. Spatial overlap (D) between the segmentation methods. For these calculations, FIRST was run in Freesurfer space (on orig.mgz) and the segmentation was mapped back into native space to allow Dice computation. The dotted and the gray boxes show the spatial overlap relative to the manual tracing by Observer 1 and Observer 2, respectively. Significant differences among the three automated methods when compared to manual tracing are marked by asterisks (* P<0.01, ** P<0.001, *** P<0.0001; post-hoc Dunn's multiple comparisons test). Whiskers are set at minimum and maximum, the horizontal line marks the median, whereas box indicates the interquartile range (25-75%).

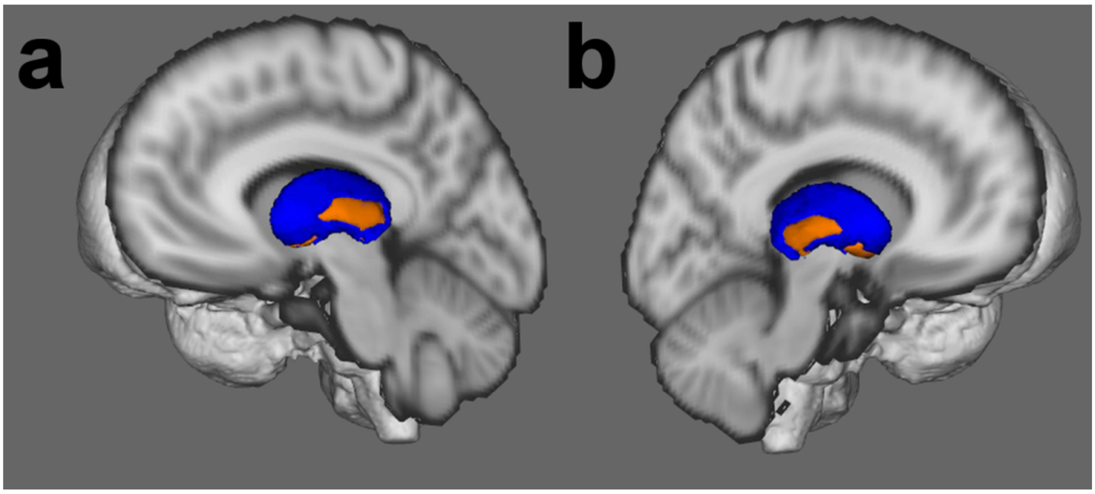

Supplementary Figure S2. Shape analyses between Freesurfer 5.3 and Observer 2 for the left **(a)** and right **(b)** putamen. Blue indicates the average shape of putamen segmentation (averaged across all the MNI transformed putamen segmentations by Freesurfer 5.3 and Observer 2 together). Orange denotes significant surface expansion of the putamen segmentation by Freesurfer 5.3 relative to the segmentation by Observer 2 ($P<0.05$, corrected for multiple comparisons).

Supplementary References

1. Jenkinson, M., Beckmann, C. F., Behrens, T. E., Woolrich, M. W. & Smith, S. M. Fsl. *Neuroimage* **62**, 782-790 (2012).
2. Winkler, A. M., Ridgway, G. R., Webster, M. A., Smith, S. M. & Nichols, T. E. Permutation inference for the general linear model. *Neuroimage* **92**, 381-397 (2014).
